# The psychoactive drug of abuse mephedrone differentially disrupts blood-brain barrier properties

**DOI:** 10.1186/s12974-021-02116-z

**Published:** 2021-03-01

**Authors:** Tetyana P. Buzhdygan, Cassidy R. Rodrigues, Hannah M. McGary, Jana A. Khan, Allison M. Andrews, Scott M. Rawls, Servio H. Ramirez

**Affiliations:** 1 Department of Pathology and Laboratory Medicine, The Lewis Katz School of Medicine at Temple University, 3500 N Broad St, Philadelphia, PA 19140 USA; 2 Center for Substance Abuse Research, The Lewis Katz School of Medicine at Temple University, Philadelphia, PA 19140 USA; 3 Shriners Hospital Pediatric Research Center, The Lewis Katz School of Medicine at Temple University, Philadelphia, PA 19140 USA

**Keywords:** Cathinones, Mephedrone, BBB, Substance abuse, Neuroinflammation

## Abstract

**Background:**

Synthetic cathinones are a category of psychostimulants belonging to the growing number of designer drugs also known as “Novel Psychoactive Substances” (NPS). In recent years, NPS have gained popularity in the recreational drug market due to their amphetamine-like stimulant effects, low cost, ease of availability, and lack of detection by conventional toxicology screening. All these factors have led to an increase in NPS substance abuse among the young adults, followed by spike of overdose-related fatalities and adverse effects, severe neurotoxicity, and cerebral vascular complications. Much remains unknown about how synthetic cathinones negatively affect the CNS and the status of the blood-brain barrier (BBB).

**Methods:**

We used in vitro models of the BBB and primary human brain microvascular endothelial cells (hBMVEC) to investigate the effects of the synthetic cathinone, 4-methyl methcathinone (mephedrone), on BBB properties.

**Results:**

We showed that mephedrone exposure resulted in the loss of barrier properties and endothelial dysfunction of primary hBMVEC. Increased permeability and decreased transendothelial electrical resistance of the endothelial barrier were attributed to changes in key proteins involved in the tight junction formation. Elevated expression of matrix metalloproteinases, angiogenic growth factors, and inflammatory cytokines can be explained by TLR-4-dependent activation of NF-κB signaling.

**Conclusions:**

In this first characterization of the effects of a synthetic cathinone on human brain endothelial cells, it appears clear that mephedrone-induced damage of the BBB is not limited by the disruption of the barrier properties but also include endothelial activation and inflammation. This may especially be important in comorbid situations of mephedrone abuse and HIV-1 infections. In this context, mephedrone could negatively affect HIV-1 neuroinvasion and NeuroAIDS progression.

## Introduction

Cathinone (2-amino-1-phenyl propanone) is the naturally occurring psychostimulant and the major active constituent of khat, the leaf of the *Catha edulis* bush that has been chewed recreationally in East Africa and Middle East for centuries. Cathinone is the b-keto analog of amphetamine (1-phenylpropan-2-amine) and modifications to the basic structure of the cathinone result in cathinone derivatives, such as 4-methylmethcathinone or mephedrone. Mephedrone (4-MMC; 1-(4-methylphenyl)-2-methylaminopropane-1-one) was first synthesized in 1929 and is one of the most potent derivatives of the cathinone [1]. Mephedrone abuse became an emerging public health problem in late 2000s, affecting adolescent and young adult users worldwide. Growing number of reports on the synthetic cathinone abuse highlights the serious physical and psychological risks. Similar to other phenylethylamines, mephedrone acts as a dopamine (DA) and serotonin (5-HT) transporter substrate, disrupting vesicular storage and dramatically increasing dopamine and serotonin levels [1–4], thus eliciting powerful psychostimulant, enactogenic, and hallucinogenic effects. Potency, low cost, and wide availability of mephedrone made it desirable substitute to more expensive illegal drugs that share similar mechanism of action, such as amphetamine, ecstasy (MDMA), and methamphetamine (METH) [5, 6]. Numerous reports state that mephedrone is also used as a partial filler of ecstasy preparations [7], thus drastically increasing risk of overdose and adverse side effects.

Mephedrone was reported to significantly increase the levels of lipid peroxidation and expression of antioxidant enzymes (such as superoxide dismutase, catalase, and glutathione peroxidase) throughout brain regions of mice and rats [8, 9]. Mephedrone treatment in vitro causes a concentration-dependent death of murine neurons [10]. However, mephedrone toxicity to the brain endothelium has not been addressed yet.

The blood-brain barrier (BBB) is essential for the function of neuronal networks, and popular drugs of abuse negatively impact BBB integrity and cause endothelial activation [11–13]. METH and MDMA, well-characterized drugs of abuse with close structural and mechanistic similarities to mephedrone, were both reported to disrupt the BBB in vivo as evidenced by brain edema and leakage of Evans blue dye in the brain parenchyma [14–17]. In vitro, METH treatment results in dose-dependent decrease in transendothelial electrical resistance and increased passage of fluorescein isothiocyanate (FITC)-labeled dextran [18–20]. Loss of BBB integrity is often attributed to the abnormalities of tight junctions and neuroinflammation. METH was shown to decrease the expression of zona occludens 1 (ZO-1), occludin, and claudin-5 both in vivo and in vitro [18–21], activate MMP9 [21, 22], and induce secretion of proinflammatory cytokines [18]. Similarly, MDMA was reported to increase expression of proinflammatory cytokines [23, 24] and reactive oxygen species [25]. Notably, a major pathological characteristic of mephedrone and METH overdose is brain edema [26, 27]; however, the mechanism of mephedrone-induced BBB damage has not been addressed yet.

The objective of this study was to investigate the effects of mephedrone on the integrity and function of the BBB. Using in vitro models of the BBB, we found that mephedrone decreases transendothelial electrical resistance (TEER) and increases paracellular permeability of the endothelial barrier. Analysis of proinflammatory and angiogenic responses presented in this report offers possible mechanisms of mephedrone-induced BBB disruption.

## Materials and methods

### Reagents

Racemic mephedrone (50:50 ratio of R-mephedrone to S-mephedrone) was prepared by and obtained from Fox Chase Chemical Diversity, Inc. (Doylestown, PA) as previously described [28].

### Endothelial cell culture and TAK242 treatment

All experiments used primary human brain endothelial cells derived from fetal brain tissue. Fetal human brain microvascular endothelial cells (hBMVEC) were isolated as described [29]. Cells were grown on rat-tail collagen I-coated flasks (BD Biosciences) in complete medium (EBM-2 supplemented with EGM-2MV SingleQuots, Lonza, Cat No CC-3156 and CC-4147) in an incubator set to 37 °C, 5% CO_2_, and 100% humidity.

For TAK242 treatment, complete cell culture medium was changed to growth factors-free/serum-free medium for overnight, followed by treatment with 100 nM TAK242 (Sigma, Cat No 614316) for 3 h.

### Cell viability assay

The LIVE/DEAD viability/cytotoxicity assay (Life Technologies) was used to evaluate mephedrone toxicity to hBMVEC cell. Briefly, hBMVEC cells were seeded on a sterile 96-well plate at 1 × 10^4^ cells per well and grown to confluency. Confluent cells were treated with 1 μM, 10 μM, or 100 μM mephedrone in growth factor-free medium for 96 h. Two hundred microliters of 1 μM calcein-AM was added to each well and incubated for 30 min at room temperature. Data was acquired at excitation and emission wavelengths of 495/515 nm and presented as ratio of live cells in mephedrone-treated wells normalized to control (untreated) cells.

### Cell proliferation assay

To evaluate the effect of mephedrone on the cell proliferation, hBMVEC cells were seeded on 5 sterile 96-well plates at 5 × 10^3^ cells per well and incubated at room temperature for 30 min to allow cells attachment and even distribution within the well. After 30 min, cells were treated with 10 μM mephedrone in growth factor-free medium and placed in the incubator set to 37 °C, 5% CO_2_, and 100% humidity. Every 24 h, 200 μL of 1 μM calcein-AM was added to the wells in one plate and incubated for 30 min at room temperature. Data was acquired at excitation and emission wavelengths of 495/515 nm and presented as fluorescence intensity. Experiment continued for 5 days at which point confluency in both control and experimental plates was reached.

### Electric cell-substrate impedance sensing assay

Real-time changes of transendothelial electrical resistance were monitored using the electric cell-substrate impedance sensing (ECIS) ZTheta 96 Well Array Station (Applied Biophysics). ECIS was recorded using the multiple frequency/time (MFT) option to continuously monitor changes in impedance over spectrum of frequencies (400 Hz to 48,000 Hz). 96W20idf PET arrays were incubated with 10 mM cysteine solution to stabilize gold electrodes followed by coating with rat-tail collagen type 1. Cells were plated at the density of 10,000 cells per each well with one well left cell-free for model purpose and grown until confluent monolayer and functional barrier were formed as indicated by stable resistance > 600 Ohm at 4000 Hz and capacitance < 20 mA at frequency 400 Hz. For the growing phase (5 to 7 days) cells were maintained in complete medium with 50% of medium changed every second day. After the confluent and functional monolayer was formed, mephedrone at various concentrations (1 μM, 5 μM, 10 μM) was added to quadruplicated wells and the recording continued for 48 h. Intercellular barrier resistance component was extracted using the Rb (barrier resistance) modeling function of the ECIS software.

### Paracellular permeability assay

To evaluate the paracellular permeability, cells were seeded at the density of 10,000 cell per collagen I-coated Transwell insert (pore size 0.4 μm, diameter 0.33 cm^2^, Corning) in the 200 μL of EGM2-MV medium. Basolateral chambers were filled with 500 μL of medium. Medium was changed every 3 days. After confluent monolayer was formed, hBMVEC monolayers were serum starved for 1 h and then incubated with growth factor-free/serum-free medium with 10 μM mephedrone or 50 ng/mL tumor necrosis factor alpha (TNFα) for 30 min. FITC-conjugated dextran (Sigma) was added to the apical chamber to the final concentration of 2 mg/mL, and 1, 3, or 24 h later, medium from the basolateral chamber was carefully removed and fluorescence was measured at 525 nm using a SpectraMax M5e (Molecular Devices). Apparent permeability coefficient (*P*_app_) was determined using the following equation: *P*_*app*_=$\frac{\left(\frac{\Delta  Q}{\Delta  t}\right)}{A{C}_0}$, where $\left(\frac{\Delta  Q}{\Delta  t}\right)$is the steady-state flux of tracer (mg/s), *A* is the surface area of the permeable insert (0.33cm^2^), and *C*_0_ is the initial concentration in the donor chamber (in mg/mL) as described [30].

### Quantitative real-time PCR

To examine the concentration of mRNA, total ribonucleic acid (RNA) was extracted using TRIzol and PureLink RNA extraction reagents (Invitrogen). cDNA was synthesized with 500 ng of RNA in 20 μL reaction mix using HighCapacity cDNA Reverse Transcriptase kit (Applied Biosystems). qRT-PCR was performed using TaqMan Universal 2x Master Mix (Thermo Scientific) and human tissue inhibitor matrix metalloproteinase 1 (*TIMP-1*) (Hs01092512), *MMP3* (Hs00968305), *MMP9* (Hs00957562), *MMP12* (Hs00159178), and FAM-labeled probes. *18S* rRNA (Cat No 4352930) was used as an internal control. Gene expression levels were analyzed using 2^−ΔΔCt^ algorithm.

### Protein electrophoresis and immunoblotting

Confluent cell monolayers were briefly rinsed with PBS and processed for cell fractionation using ProteoExtract Subcellular Proteome Extraction kit (EMD Millipore) as per manufacturer’s protocol. Obtained fractions were subjected to 10% Bis-Tris polyacrylamide gel electrophoresis in MOPS buffer under denaturing conditions and transferred to a 0.45 μm PVDF membrane. Membrane was blocked with Odyssey blocking buffer in phosphate-buffered saline (Li-Cor Biosciences, Cat No 927-40000) for 1 h at room temperature. Blocked protein blot was incubated with affinity-purified rabbit anti-claudin-1 (1:1000, Invitrogen Cat No 51-9000), rabbit anti-claudin-5 (1:1000, Abcam, Cat No 131259), rabbit anti-occludin (1:500, Invitrogen, Cat No 71-1500), rabbit anti toll-like receptor 4 (TLR4) (1:500, Invitrogen, Cat No 48-2300), and membrane fraction WB cocktail (1:200, Abcam Cat No 140365) in Odyssey blocking buffer supplemented with 0.05% Tween-20 at 4 °C overnight, followed by incubation with IRDye 800CW goat anti-rabbit and IRDye 680RD goat anti-mouse secondary antibodies (Li-Cor) diluted 1:10,000 in Odyssey blocking buffer supplemented with 0.05% Tween-20 and 0.01% SDS at room temperature for 45 min. Protein blot was visualized with Odyssey Imaging System (Li-Cor). Band intensities were quantified using ImageJ software (NIH, Bethesda). Data is presented as the relative intensity of mephedrone as function of untreated bands normalized to the GAPDH (cytoplasmic fraction) or NaK-ATPase (membrane fraction).

### Angiogenic factors ELISA

To examine the concentration of growth factors secreted by hBMVEC, cells were grown to confluency and treated with 10 μM mephedrone in growth factor-free medium for 24 h. Cell culture supernatant was briefly centrifugated (5 min at 2000*g*) to pellet cell debris and then analyzed using Human PDGF BB Elisa kit (Invitrogen Cat No BMS2071) or vascular endothelial growth factor A (VEGF-A) Quantikine Elisa kit (RnD Systems Cat No DVE00) as described in the manufacturer’s protocol.

### MMP9 activity assay

To examine the activity of MMP9 secreted by hBMVEC, cells were grown to confluency and treated with 10 μM mephedrone in growth factor-free medium for 24 h. Cell culture supernatant was briefly centrifugated (5 min at 2000*g*) to pellet cell debris and then analyzed using Human Active MMP-9 Fluorikine E kit (RnD Systems, Cat No F9M00) as described in the manufacturer’s protocol.

### Proinflammatory cytokine expression panel

To examine the concentration of proinflammatory cytokines secreted by hBMVEC, cells were grown to confluency and then treated with 10 μM mephedrone in growth factor-free medium for 24 h. Cell culture supernatant was briefly centrifugated (5 min at 2000*g*) to pellet cell debris and then analyzed using V-PLEX Proinflammatory Panel I Human kit (MesoScale Diagnostics Cat No K15049D) as described in the manufacturer’s protocol.

### Nuclear factor kappa B (NF-κB) activation assay

To examine the activation of NF-κB subunits, hBMVEC were grown to confluency, medium was changed to growth factor-free/serum-free medium, and mephedrone was added at 10 μM final concentration for 24 h. For TLR4 inhibition experiment, cells were pre-treated with 100 nM TAK242 in growth factor-free/serum-free medium for 3 h, followed by mephedrone treatment. Confluent cell monolayers were briefly rinsed with PBS and processed for cell fractionation using ProteoExtract Subcellular Proteome Extraction kit (EMD Millipore) as per the manufacturer’s protocol. Nuclear extracts were analyzed using TransAM NF-κB Activation Assays (Active Motif, Cat No 43296) as per the manufacturer’s protocol. For analysis, 10 ug of nuclear extract was loaded per each well.

### Immunofluorescence staining

Assessment of p65 translocation to the nucleus was performed by indirect immunofluorescence. hBMVEC were grown to 70% confluency, treated with 10μM mephedrone in growth factor-free /serum-free medium for 24 h and fixed/permeabilized in 50%/50% methanol/acetone for 15 min at – 20 °C, blocked in 1% BSA in PBS and incubated with rabbit anti-NF-κB p65 (Cell Signalling, Cat No 8242, 1:500) antibodies for 16 h at 4 °C. Secondary antibodies AlexaFluor 488-conjugated goat anti-rabbit were applied for 2 h at room temperature. Cells were then incubated with DAPI nuclear stain (4′,6-diamidino-2-phenylindole, Invitrogen), rinsed with PBS, and coverslips were mounted using ProLong Antifade reagent (Invitrogen). Immunofluorescence was visualized using a Nikon Eclipse 80i fluorescent microscope (Nikon, Tokyo, Japan).

### Flow cytometry

Cells were washed with calcium- and magnesium-free PBS, detached using accutase and then pelleted by centrifugation at 1000 rpm for 5 min. Cells were resuspended in fixation buffer (eBioscience/Thermo Fisher) and incubated for 30 min. Following fixation, cells were washed with flow cytometry buffer (5% FBS with 0.1% sodium azide) and pelleted again. Cells were resuspended in 100 μL of flow cytometry buffer and anti-intercellular adhesion molecule 1 (ICAM-1) (FITC), anti-VCAM (APC), and anti-CD31/platelet endothelial cell adhesion molecule (PECAM-1) (Pacific Blue) pre-conjugated primary antibodies for 30 min. Cells were then washed, pelleted, and resuspended in flow cytometry buffer for flow cytometric analysis. Ten thousand events for each sample were acquired with a FACS BD Canto II flow cytometer (BD Biosciences) and analyzed with FlowJo software (Tree Star, Ashland, OR, USA).

### Statistical analysis

**Fig. 1:**
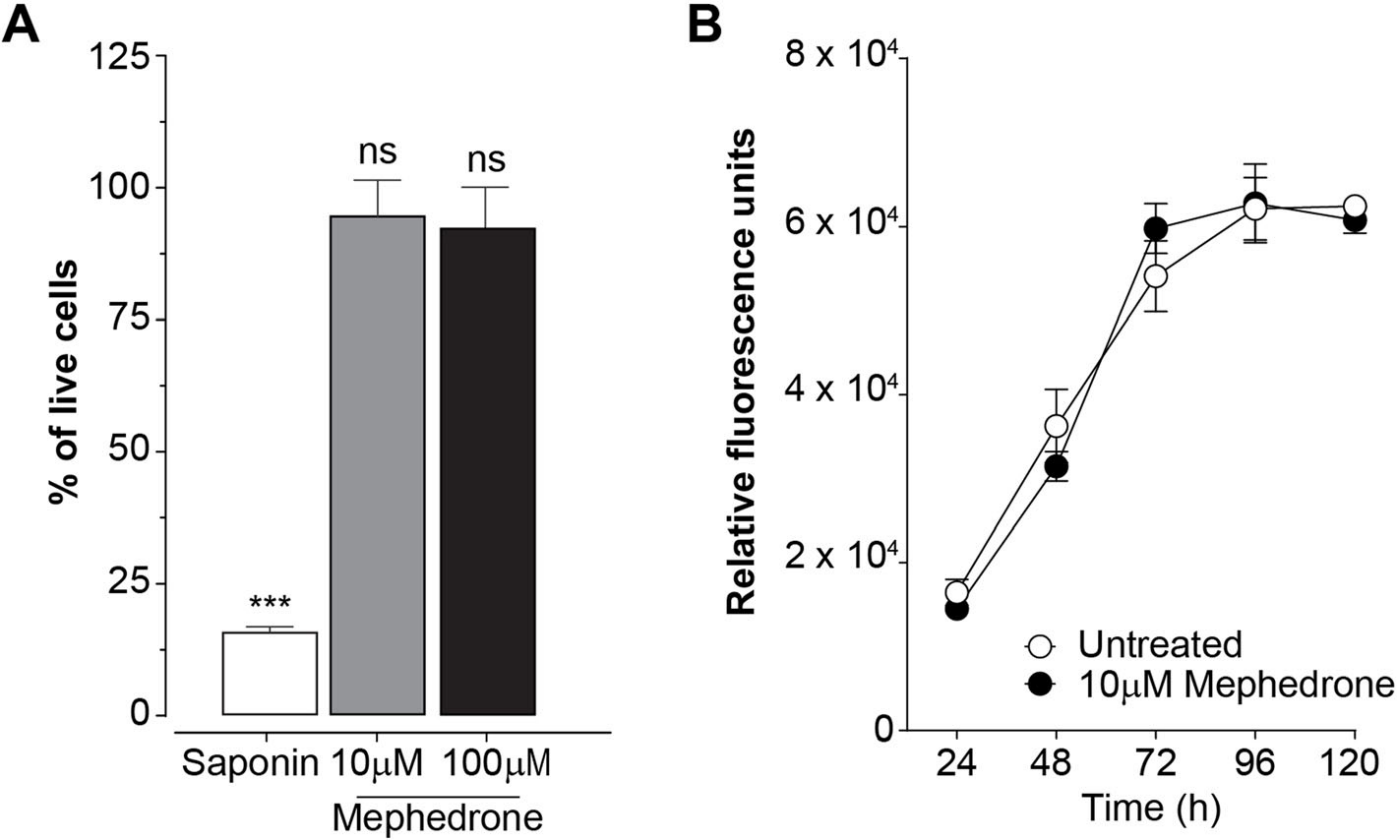
Mephedrone does not affect cell growth and viability of hBMVEC. **a** Treatments (96 h) with 10 μM or 100 μM mephedrone did not cause significant toxicity or cell death compared with untreated controls. Saponin (sap) was used as a positive control. Data is presented as the mean number of live cells ± SEM. Experiments were independently performed three times. Within each individual experimental set, primary cells from 3 different donors were used (*n* = 9). **b** Curves represent cell proliferation at 0 h through 120 h. Persistent exposure to 10 μM mephedrone did not change rate of cell growth. Average number of live cells was determined using Cell Viability Assay (Promega). Data is presented as the mean number of live cells ± SEM. Experiments were independently performed three times. Within each individual experimental set, primary cells from 3 different donors were used (*n* = 9)

**Fig. 2:**
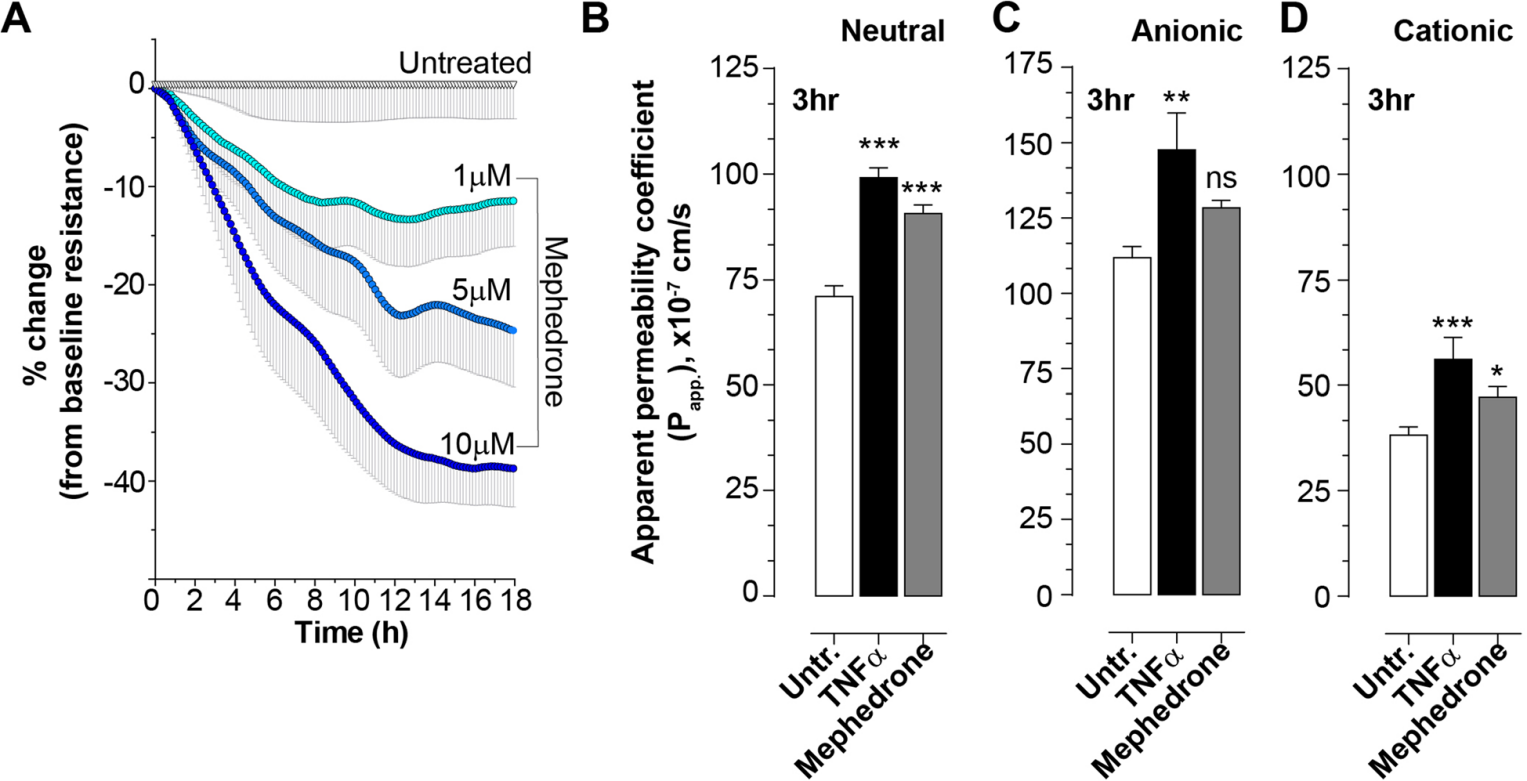
Mephedrone compromises barrier properties of hBMVEC. **a** Barrier electrical resistance was modelled based on continuous cell-substrate impedance readings recorded at 6 frequencies (400 Hz–48 kHz) every 6 min for the duration of the time shown. Endothelial monolayers were treated with 1 μM, 5 μM, or 10 μM mephedrone or left untreated to serve as a baseline. Treatments were initiated at 0 timepoint. Each data point is represented as the percentage of the mean value ± SEM. Experiments were independently performed three times. Within each individual experimental set, primary cells from four different donors were used (*n* = 12). **b**–**d** Barrier permeability of small molecular tracer was modelled using FITC-conjugated dextrans (3 kDa) of various charges: neutral (**b**), anionic (**c**), and cationic (**d**). Endothelial monolayers were treated with 10 μM mephedrone, 100 ng/mL TNFα, or left untreated to serve as a baseline. Each data point is represented as the apparent permeability coefficient (*P*_app_, mean value ± SEM). Experiments were independently performed three times. Within each individual experimental set, primary cells from 6 different donors were used (*n* = 18)

**Fig. 3:**
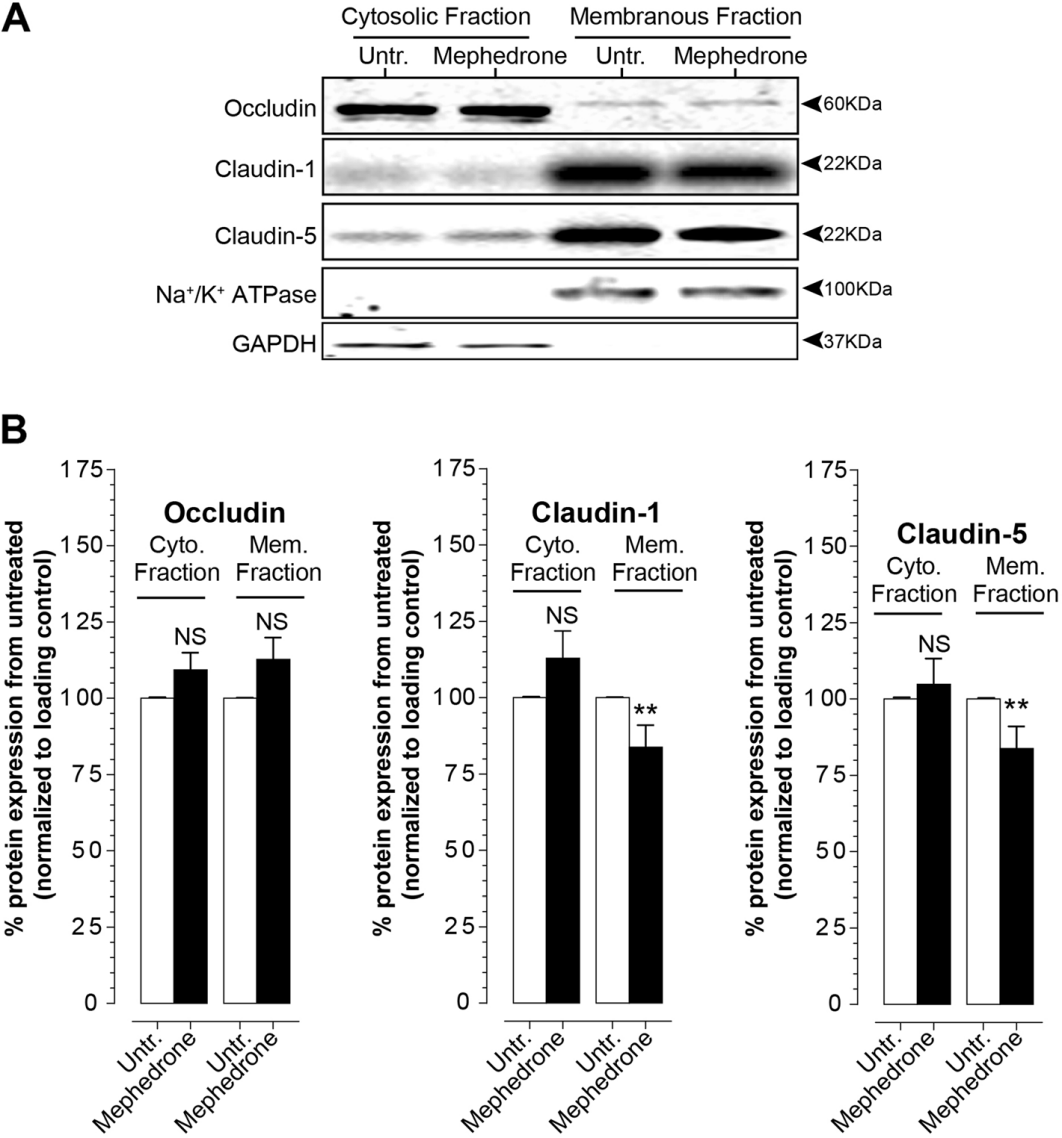
Mephedrone affects tight junction protein expression and localization in hBMVEC*.* **a** Representative immunoblots of occludin, claudin-1, and claudin-5 from cytosolic and membrane fractions of endothelial cells treated with 10 μM mephedrone for 24 h. **b** The plots represent densitometry analysis of the target band intensity normalized to the GAPDH (cytosolic fraction) or NaK-ATPase (membrane fraction) band intensities and relative to the untreated control. The results are shown as the percentage of the mean value ± SEM. Experiments were independently performed three times. Within each individual experimental set, primary cells from four different donors were used (*n* = 12)

**Fig. 4:**
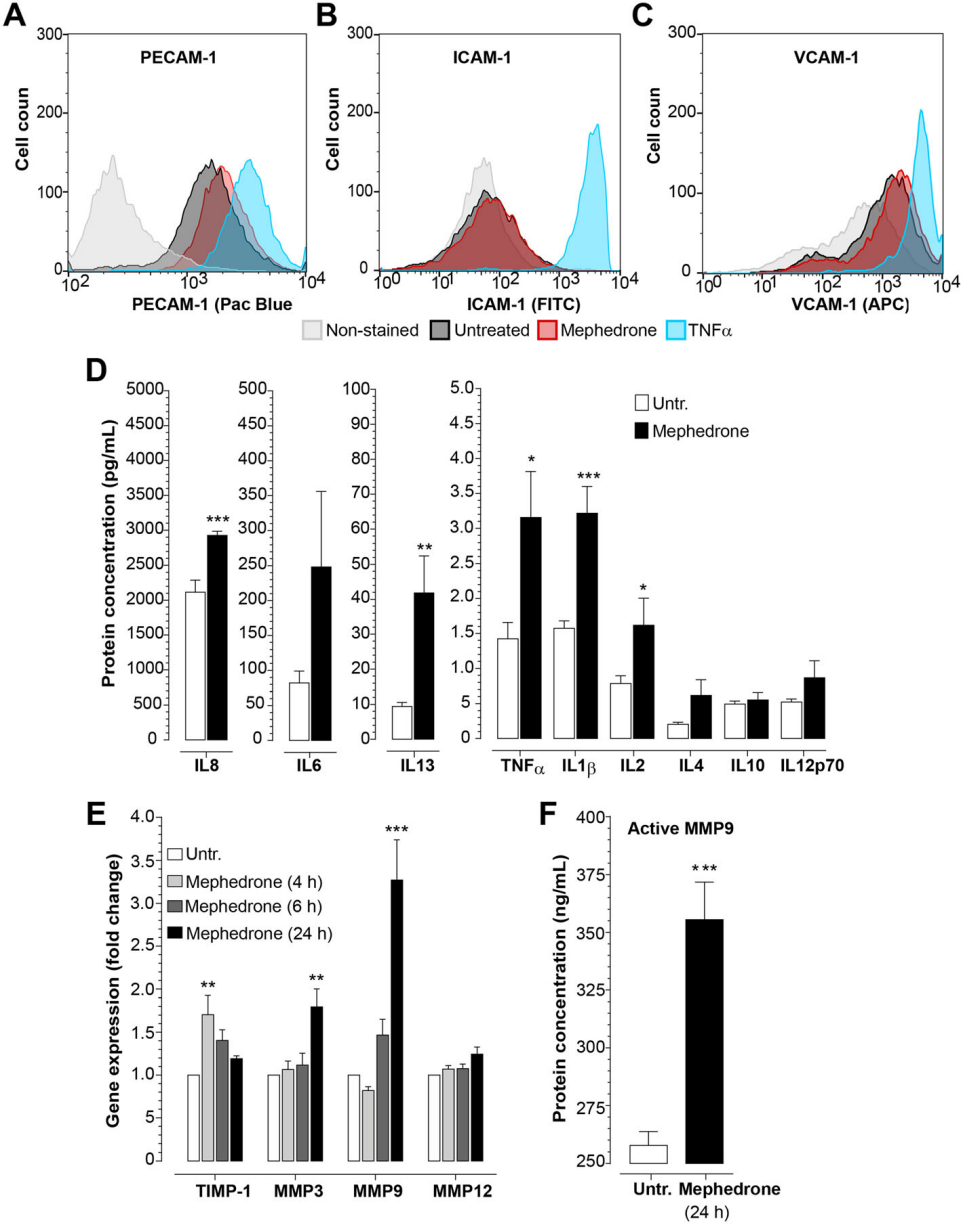
Mephedrone induces inflammatory activation of hBMVEC. **a**–**c** hBMVECs were treated with 10 μM mephedrone for 2 h and analyzed by flow cytometry. Histograms show the expression of adhesion molecules PECAM-1, ICAM-1, and VCAM-1 in response to mephedrone, which was statistically significant only for PECAM-1 at 2 h. Experiments were independently performed three times. Within each individual experimental set, primary cells from three different donors were used (*n* = 9). **d** hBMVEC were treated with 10 μM mephedrone for 24 h, and levels of cytokines were analyzed by V-PLEX assay. Levels of IL-1β, IL-2, IL-8, IL-13, and TNFα were significantly upregulated by the treatment. Data shown as the mean concentration ± SEM expressed in pg/mL. Experiments were independently performed three times. Within each individual experimental set, primary cells from three different donors were used (*n* = 9). **e** *TIMP-1*, *MMP3*, *MMP9*, and *MMP12* mRNA expression in hBMVEC treated with 10 μM mephedrone for 4, 6, or 24 h were determined using qRT-PCR. Data shown as fold change relative to untreated control and normalized to the housekeeping gene (*18S*). Experiments were independently performed three times. Within each individual experimental set, primary cells from three different donors were used (*n* = 9). **f** MMP9 enzyme activity in the culture medium of endothelial cells treated with 10 μM mephedrone for 24 h were determined using Human Active MMP9 Fluorokine assay. Data shown as mean ± SEM and expressed in pg/mL of active enzyme. Experiments were independently performed three times. Within each individual experimental set, primary cells from three different donors were used (*n* = 9)

**Fig. 5:**
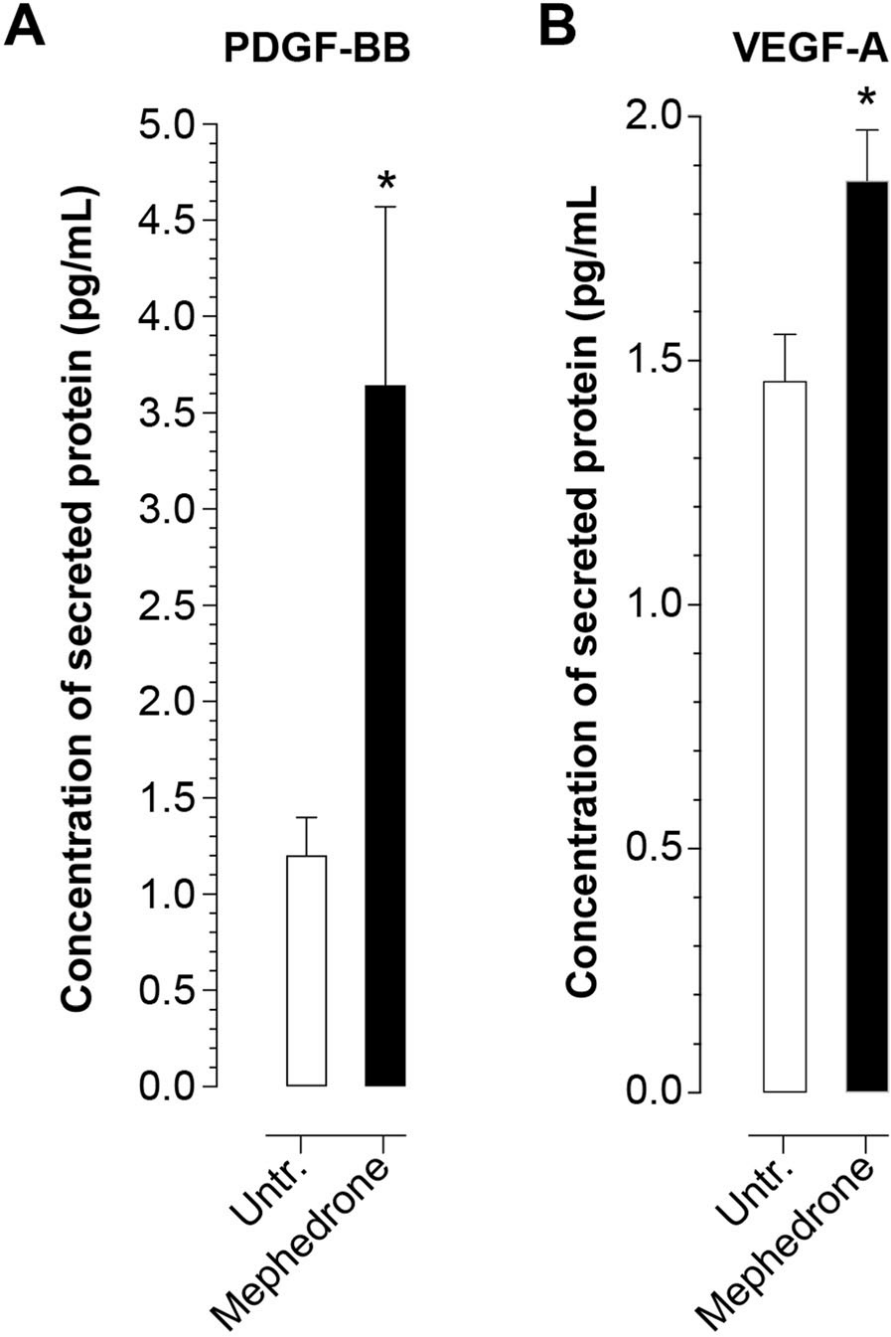
Mephedrone induces angiogenic mechanisms. ELISA was used to measure the concentrations of secreted angiogenic growth factors: PDGF-BB (**a**) and VEGF-A (**b**) in cell culture medium of hBMVEC treated with 10 μM mephedrone for 24 h. Data shown as the mean concentration ± SEM expressed in pg/mL. Experiments were independently performed three times. Within each individual experimental set, primary cells from three different donors were used (*n* = 9)

**Fig. 6:**
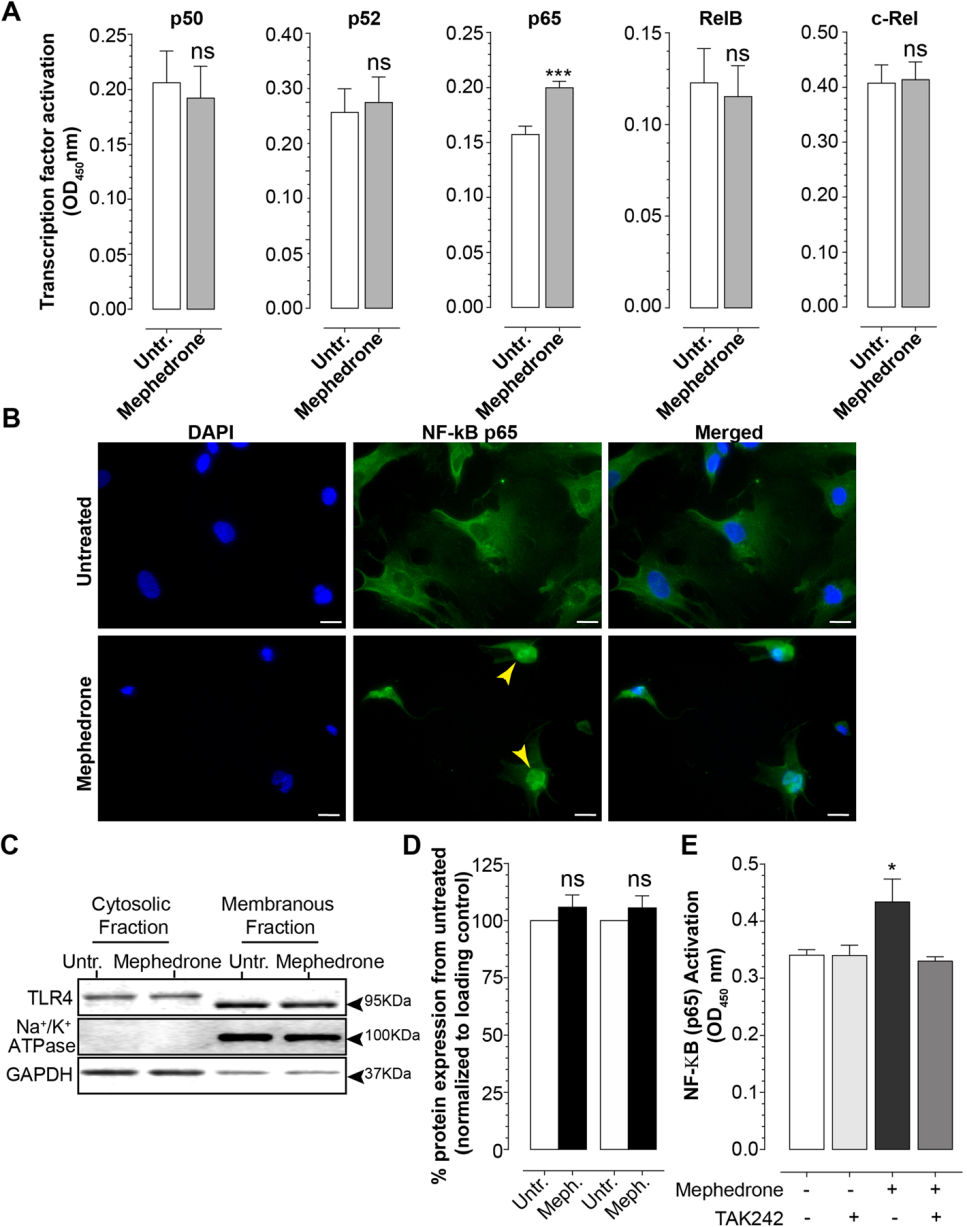
Mephedrone activates NF-κB signaling pathway. **a** TransAM Transcription Factor Activation Assay was used to measure concentrations of activated NF-κB subunits in the nuclear fractions of endothelial cells treated with 10 μM mephedrone for 24 h. Mephedrone significantly upregulates the level of nuclear p65 subunit. Data shown as mean ± SEM. Experiments were independently performed three times. Within each individual experimental set, primary cells from three different donors were used (*n* = 9). **b** Representative image of p65 nuclear translocation in endothelial cells. Scale bar is 10 μm. Representative immunoblot (**c**) and densitometry analysis (**d**) of TLR4 expression in cytosolic and membrane fractions of hBMVEC. Data shown as mean ± SEM. Experiments were independently performed three times. Within each individual experimental set, primary cells from three different donors were used (*n* = 9). **e** TransAM NF-κB p65 Activation Assay was used to measure concentrations of activated p65 in the nuclear fraction of endothelial cells treated with 10 μM mephedrone alone or pre-treated with 100 nM TAK242 for 3 h. TAK242 significantly downregulates the amount of mephedrone-activated p65 subunit. Data shown as mean ± SEM. Experiments were independently performed three times. Within each individual experimental set, primary cells from three different donors were used (*n* = 9)

The experiments were independently performed multiple times (at least three times for all the data shown) to allow statistical analyses. Within each individual experimental set, primary cells from at least 3 donors were used. Shapiro-Wilk test was used to test for normal distribution of data. All data sets presented in this report passed normality sets (*α* = 0.05). The Student *t* test was used to analyze two groups (Figs. 1b, 3b, 4d and f, 5a and b, and 6a and d). One-way ANOVA with post-hoc Dunnett’s test was used when multiple group comparisons were performed against a reference control (Figs. 1a, 2b–d and 4e). One-way ANOVA with post-hoc Tukey’s test was used to compare mean of each data set with the mean of every other data set (Fig. 6d). Repeated measure one-way ANOVA with post-hoc Dunnett’s test was used to analyze data for Fig. 2a. Results are expressed as the mean ± SEM with differences considered significant at *p* < 0.05. The data collected was analyzed using Prism v6.0 (GraphPad Software, San Diego, CA, USA).

## Results

### Mephedrone does not induce cellular toxicity in human brain endothelial cells

To date, no published information is available regarding whether mephedrone impacts viability of human endothelial cells. Therefore, to assess the cell toxicity of mephedrone, hBMVECs were exposed to increasing concentrations of mephedrone and analyzed using the Calcein-AM Live/Dead Cytotoxicity assay (Promega). The assay utilizes the cell-permeable non-fluorescent acetomethoxy-derivative of calcein (calcein-AM), which readily enters living cells. Once in the cytosol, cellular ferment esterase removes AM groups, allowing calcein to bind calcium and preventing it from leaving the cell. Calcein released from AM become a light activated fluorophore. Figure 1a shows the results of hBMVEC viability after exposure of two different concentrations of mephedrone (10 μM and 100 μM) for 96 h. Of note, 10 μM is the average concentration of mephedrone in the plasma after drug intake [30]. The results are represented as percent of untreated cells that were fluorescently positive (calcein). The mild detergent, saponin, was used as a positive control. Our results showed no evidence of cytotoxicity with the concentrations tested, including the 100 μM concentration, which is 10-fold higher than mephedrone level detected in the plasma of overdose patient. The percent of viable cells left untreated was 99.25% ± 1.92% (mean ± SEM), treated with saponin 15.67% ± 0.37% (*p* < 0.001), 10 μM mephedrone 95.13% ± 2.47% (ns), and 100 μM mephedrone 92.88% ± 2.78% (ns). In the absence of toxicity, the ability for mephedrone to induce cell growth or proliferation was investigated. To this end, analysis of cell growth curves of brain endothelial cells with and without mephedrone was performed. As shown in Fig. 1b, the growth rate of brain endothelial cells was not inhibited upon exposure to mephedrone with the growth curve showing a similar rate to the untreated condition. Overall, these analyses did not reveal that mephedrone impacts the viability or proliferation of primary hBMVECs.

### Mephedrone induces impairment of brain endothelial barrier integrity

Since mephedrone had no observable effects on the viability or proliferation of brain endothelial cells, the next set of experiments focused on evaluating whether mephedrone impacts barrier integrity and function. The main feature that distinguishes brain vasculature endothelium from those in the periphery is the abundant presence of tight junctions formed between adjacent cells. Tight junctions form the physical barrier of the BBB, preventing the free paracellular flux of ions and small molecules. Two main parameters used to assess the integrity of the BBB and tight junctions are transendothelial electrical resistance (TEER) and paracellular permeability (PP). TEER is the function of three factors: (1) the number of tight junctional strands, (2) the specific resistance of the strands (which is determined by their protein composition and is regulated by variety of factors), and (3) the number of open channels. PP is determined by two distinct factors: (1) the number of open channels and (2) the time (and probability) of each channel to be open in every single moment. It is important to note that TEER and PP reflect different functional properties of the tight junctions and, therefore, do not necessarily perform in parallel. The drop in TEER clearly indicates the BBB opening, but does not linearly correspond to the magnitude in paracellular permeability, which is why both assays should be performed to fully evaluate the BBB function. Therefore, to characterize mephedrone-induced disruption of the endothelial barrier, we examined both parameters in our study. TEER measurements were acquired as described in the “Materials and methods” section. Cells were plated on interdigitated electrode arrays (96W 20idf from Applied BioPhysics) with a surface area of 3.98 mm^2^ measuring continuously the resistance from ~ 8000 cells. The baseline resistance measured at 4000 Hz was 670 Ohms for donor A, 590 Ohms for donor B, and 780 Ohms for donor C. As shown in Fig. 2a, at 18 h, the electrical resistance of the monolayers containing 1 μM mephedrone showed an average decrease of − 11.5% ± 4.60% (mean ± SEM, *p* < 0.001) from baseline. At 5 μM mephedrone, resistance values showed an average decrease of − 24.7% ± 5.76% (*p* < 0.001) whereas 10 μM mephedrone was even greater at − 38.7% ± 3.94% (*p* < 0.001).

To determine how mephedrone treatment alters the flux of molecules across the tight junctions, paracellular permeability assays were performed. In order to place focus entirely on the paracellular passage (“leak pathway”) through the tight junctions rather than via transcytosis or transendothelial channel routes, these permeability assays utilized a smaller molecular weight tracer bearing neutral charge (3k Da FITC-conjugated dextran). At early (1 h) and late (24 h) timepoints, mephedrone treatment did not significantly changed paracellular permeability (data not shown). However, at 3 h, *P*_app_ for untreated was 71.03 ± 2.49, for TNFα 99.15 ± 2.3 (*p* < 0.001), and for 10 μM mephedrone 90.68 ± 2.02 (*p* < 0.001) (Fig. 2b). The results suggest that mephedrone transiently increases the passive paracellular passage of the small molecules which is also a function of time.

To further examine whether mephedrone induces BBB disruption by neutralizing the net negative charge of the endothelium, an anionic 3 kDa FITC-dextran sulfate and a cationic 3 kDa FITC-diethylaminoethyl (DEAE) dextran were also used. Thus, if mephedrone disrupts the negative charge of the endothelium, then the changes in the permeability for anionic and cationic tracers should be the same. However, if the negative charge of the endothelial cells is preserved, then permeability of the cationic tracer would be greater than that of the anionic tracer. Again, at early (1 h) and late (24 h) timepoints, no significant differences in permeability of cationic or anionic tracers in mephedrone-treated groups were observed (data not shown). Figure 2d shows the permeability of cationic 3 kDa FITC-dextran at 3 h; *P*_app_ for untreated was 38.17 ± 1.9, for TNFα 56.12 ± 5.25 (*p* < 0.001), and for 10 μM mephedrone 47.3 ± 2.4 (*p* < 0.05). Figure 2c shows the permeability of hBMVECs to the anionic 3 kDa FITC-dextran sulfate at 3 h; *P*_app_ for untreated was 96.36 ± 3.76, for TNFα 147.6 ± 12.36 (*p* < 0.01), and for 10 μM mephedrone 128.4 ± 2.5 (ns). These results suggest that mephedrone treatment of the cerebral endothelial cells results in charge-dependent endothelial barrier disruption.

### Mephedrone alters tight junction protein expression and localization

To determine whether the changes to TEER and permeability can be explained by the status of the tight junction complex, western blots for occludin, claudin-1, and claudin-5 were performed. As can be seen in Fig. 3a, hBMVECs were exposed to mephedrone at 10 μM for 24 h and both membrane and cytosolic cellular fractions were separated. Bands intensities were normalized to GAPDH (for cytosolic) and to sodium-potassium adenosine triphosphatase (NaK-ATPase; for membrane fraction). Occludin was detected as a low molecular mass species (55 kDa) in soluble protein fractions (cytosolic) and a high molecular mass species (60 kDa) in insoluble protein fractions (membrane fraction). Higher molecular weight of membrane-bound occludin is likely attributed to the post-translational modification of the C terminus that contains apo-lateral membrane targeting signal. Occludin in both fractions showed little change in expression (data shown as mean ± SEM), 109.35% ± 5.58% (*p* = 0.108, in cytosolic) and 112.7% ± 7.20 (*p* = 0.092, in membrane) when compared to control. Analysis of claudins in cytosolic fraction yields the following results: for claudin-1 106.70% ± 13.20% (*p* = 0.402) and for claudin-5 104.79% ± 8.44% (*p* = 0.582). Unlike the cytosolic, the membranous fraction showed a decrease of 88.00% ± 1.915% (*p* < 0.001) for claudin-1 and 83.85% ± 7.14% (*p* < 0.001) for claudin-5. These results suggest that mephedrone selectively regulates the structural properties of the tight junction complex by downregulating membrane-bound claudins 1 and 5, without significant effect on occludin.

### Mephedrone triggers proinflammatory response in brain endothelial cells

The BBB is the critical interface that not only regulates the entry of solutes and nutrients into the CNS but also limits immunosurveillance. Since mephedrone appears to induce changes to the physical barrier (i.e., barrier tightness, Fig. 2), it is possible that those changes are a result of endothelial activation, which is defined as the heightened expression of cell adhesion molecules (CAMs), involved in the immune cells adhesion and migration from the bloodstream to brain parenchyma. To examine, whether mephedrone induces activation of endothelial cells, we characterized expression of PECAM-1, ICAM-1, and vascular cell adhesion molecule 1 (VCAM-1) using flow cytometry. Cultures of hBMVECs were treated with mephedrone at 10 μM for 2, 6, and 24 h. TNFα was used as a positive control. PECAM-1 showed a marginal transient increase in surface expression at 2 h (128.0% ± 20.2%, *p* < 0.05 (Fig. 4a), which returned to baseline by 6 h and remained unchanged at 24 h timepoint (data not shown). ICAM-1 and VCAM-1 were unchanged at all timepoints. Surface expression of ICAM-1 and VCAM-1 at 2 h after treatment is shown in Fig. 4b and c, respectively.

To examine whether mephedrone induces release of proinflammatory cytokines by hBMVEC, confluent cell monolayers were treated with 10 μM mephedrone for 24 h and cell culture supernatants were analyzed using the V-PLEX Proinflammatory Panels 1 Human kit (MesoScale Diagnostics), which includes interleukin (IL)-1β, IL-2, IL-4, IL-6, IL-8, IL-10, IL-12p70, IL-13, and TNFα. Out of 9 analytes, the expression of the following cytokines was significantly increased in mephedrone-treated cells (Fig. 4d). Fold changes from untreated cells (expressed in difference between means ± SEM, pg/mL) were as follows: TNFα (1.73 ± 0.69, *p* = 0.026), IL-1β (1.64 ± 0.39, *p* = 0.001), IL-2 (0.83 ± 0.38, *p* = 0.047), IL-8 (809.1 ± 181.8, *p* = 0.006), and IL-13 (32.4 ± 10.67, *p* = 0.089). For IL-4, IL-6, IL-10, and IL-12p70, there was no statistical significance when compared to the untreated control.

It is well established that the activation of matrix metalloproteinases (MMPs) induces barrier dysfunction and leakage [31–34]. MMPs constitute a family of zinc- and calcium-dependent endopeptidases that function in the breakdown of extracellular matrix (ECM) during normal physiological and pathological processes. To examine whether mephedrone affects expression of MMPs, hBMVEC were treated with 10 μM mephedrone, and after 4 h, 6 h, or 24 h, the mRNA was isolated and analyzed by qRT-PCR. The results (Fig. 4e) show that 24 h of mephedrone treatment upregulates *MMP3* and *MMP9* mRNA expression 1.8-fold (*p* < 0.001) and 3.27-fold (*p* < 0.001), respectively, while *MMP12* mRNA was not affected. Noteworthy, *TIMP-1* mRNA which is the natural inhibitor of MMP3 and MMP9, was significantly upregulated at an early time point of mephedrone treatment (1.7-fold, *p* < 0.005); however, within the course of treatment, *TIMP-1* mRNA concentration went down allowing MMP9 activation (Fig. 4e and f).

While the concentrations of newly synthesized MMPs are regulated at the levels of transcription, the proteolytic activities of MMPs are controlled either by the activation of pro-enzymes or zymogens or by the inhibition of active enzymes by endogenous inhibitors (such as TIMPs). MMP-9 degrades components of the ECM and a variety of non-ECM molecules such as proteins of tight junctional complex. Among other MMPs, MMP9 is particularly involved in the pathological opening of BBB; therefore, MMP9 enzyme activity was further examined using the Human Active MMP9 Fluorokine E assay by R&D Systems. Active MMP9 from samples was captured by specific monoclonal antibodies followed by addition of fluorogenic substrate linked to quencher molecule. In this assay, the active MMP9s cleaves the peptide linker between the fluorophore and its quencher allowing a fluorescent signal to be detected that is proportional to the enzyme activity in the sample. Results are calibrated against highly purified CHO cell-expressed recombinant human MMP9 provided as the standard and expressed in pg/mL of active MMP9. In Fig. 4f, the significant activation of MMP9 in hBMVEC treated with 10 uM mephedrone for 24 h is observed when compared to untreated controls (355 ± 16.27 pg/mL vs 257.7 ± 5.9 pg/mL, mean ± SEM, *p* = 0.0002).

### Mephedrone activates expression of angiogenic factors

A possible explanation of mephedrone-induced changes to hBMVECs could be the activation of the angiogenic program since other drugs of abuse such as cocaine have been shown to induce expression of VEGF-A and increase vessel density in the brain [35]. Brain endothelial cells form the BBB as a result of “barriergenesis,” a process that culminates the maturation of the cerebral endothelium. However, in inflammatory environment caused by chronic substance abuse, endothelium reactivates expression of angiogenic growth factors such as platelet-derived growth factor subunit BB (PDGF-BB), VEGF-A, or tumor growth factor beta (TGF-β) [35, 36], resulting in loss of barrier properties. We evaluated the presence of angiogenic growth factors (PDGF-BB, VEGF-A, and TGF-β) in the cell culture medium of hBMVEC treated with 10 uM mephedrone for 24 h. The results in Fig. 5 show that mephedrone-treated cells secreted significantly higher levels of PDGF-BB (3.644 ± 0.928 pg/mL, compared to the 1.202 ± 0.197 pg/mL in untreated cells, mean ± SEM, *p* = 0.04) and VEGF-A (1.869 ± 0.104 pg/mL, compared to the 1.458 ± 0.095 in untreated cells, mean ± SEM, *p* = 0.011). TGF-β concentration showed no change (data not shown). Although there was increase in the expression of angiogenic growth factors, it was not paralleled by a shift in cell proliferation rate (Fig. 1b). However, the presence of these growth factors suggests that mephedrone is promoting the angiogenic program which counteracts maintenance of barrier properties.

### Mephedrone activates NF-κB signaling in hBMVECs

Due to the central role that NF-κB plays in the upregulation of proinflammatory cytokines and matrix metalloproteinase, we then investigated whether mephedrone may activate members of the NF-κB family of transcription factors. The NF-κB family includes p65, p50, c-Rel, p52, and RelB, and their activation was evaluated using NF-κB TransAM Transcription Factor Activation Assay from Active Motif Inc. This 96-well assay contains oligonucleotides with the NF-κB consensus binding site immobilized in the plate. Prepared nuclear extracts were added to the wells, and if present, activated NF-κB homodimers and heterodimers bind to the oligonucleotides. Antibodies are then directed against specific subunits that are then detected with conjugated secondary antibodies for a luminescent readout. As shown in Fig. 6a, only nuclear p65 was significantly activated at 0.199 ± 0.006 (mean ± SEM) over untreated (0.157 ± 0.007). Other subunits did not show significant nuclear activation. To confirm that mephedrone induces nuclear translocation of p65 subunit thus activating NF-κB, hBMVEC cells were treated with 10 μM mephedrone for 24 h followed by p65 immunostaining and DAPI nuclear stain. As shown in Fig. 6b, mephedrone treatment results in substantial increase in p65 localization to the cell nuclei as compared to untreated cells.

Methamphetamine, which closely resembles chemical structure and pharmacological properties of mephedrone, was reported to activate NF-κB signaling via TLR4 receptors [37]. To delineate whether TLR4 is involved in mephedrone-induced NF-κB activation, we examined TLR4 protein expression in mephedrone-treated hBMVEC. Figure 6c and d show representative immunoblot and densitometry of cytosolic and membrane TLR4 in mephedrone-treated hBMVEC compared to untreated controls. In our study, mephedrone did not significantly affect the expression or subcellular localization of TLR4. Next, we examined whether mephedrone activates TLR4 receptor signaling to induce activation of p65 subunit of NF-κB. Figure 6e shows that pre-treatment with 100 nM TAK242 counteracts mephedrone-induced activation of p65, suggesting that TLR4 acts as a link between mephedrone and NF-κB.

## Discussion

Substance abuse of synthetic cathinones has dramatically increased over the last decade. Mephedrone is extremely reinforcing and highly addictive compound, resulting in binge self-administration that often leads to overdose. Structurally related to other psychostimulants, such as METH and MDMA, mephedrone also shares a similar mechanism of action. However, unlike methamphetamine or ecstasy, little is known about mephedrone contribution to BBB disruption, endothelial activation, and neuroinflammation.

Here, we set forth to elucidate the how mephedrone may alter the status of the BBB, in regard to the permeability, tight junction complex, endothelial activation, and inflammation. Our analysis began by evaluating whether mephedrone induced any observable impact on cellular viability. For note, METH concentrations up to 200 μM (up to 10-fold of biologically relevant levels) did not induce significant cells death in immortalized hCMEC/D3 cell line or primary hBMVEC [19, 38]. Similarly, our results showed that even 100 μM mephedrone (10-fold of biologically relevant concentration [30]) did not significantly induce cell death of hBMVEC cultures. Interestingly, methylenedioxypyrovalerone (MDPV), another member of synthetic cathinones family, was reported to significantly decrease proliferation and increase necrotic cell death in primary bovine BMVEC culture [39]. However, such results can be in part explained by the fact that investigators used mM concentrations of MDPV, which is at least 100-fold higher that used in our study.

Next, we examined the effect of mephedrone on the barrier function. One of the most reported pathological characteristics of fatalities associated with mephedrone abuse is cerebral vasogenic edema [27], caused by elevated uncontrolled passage of small molecules from the bloodstream to the brain parenchyma. Therefore, to evaluate barrier integrity, we measured the electrical resistance and paracellular permeability of hBMVECs exposed to mephedrone. After one application of mephedrone, endothelial monolayers showed a steady dose-dependent decrease in the barrier electrical resistance. The effect was highest for 10 μM mephedrone, in which 40% of basal resistance was lost during the first 12 h and then plateaued. Interestingly, all concentrations of mephedrone evoked the same temporal pattern with most of the loss in resistance occurring during the first 12 h post-insult, and the degree of the reduced resistance was dose dependent. We also show that mephedrone increases the rate of passive paracellular passage of the small molecules which is also a function of time and molecular charge. These findings highlight some of the similar effects as METH, which also induces a transient dose-dependent decrease in TEER and increased passage of FITC-labeled dextran [18–20].

To arrive at a possible explanation of the mechanism of mephedrone-induced loss of the endothelial barrier functions, we analyzed the expression and subcellular localization of the key tight junction proteins that provide physical barrier to the BBB. Transmembrane proteins claudins 1 and 5 are essential structural components of tight junctions [40] and while their deletion in vivo is embryonic lethal [41], the knock-out of claudin-1 or claudin-5 does not affect the expression status of other tight junction proteins such as occludin. Degradation or redistribution of claudins are often reported in various pathologies resulting in enhanced BBB permeability and decreased electrical resistance [42, 43]. Therefore, the mephedrone-induced loss of membrane-bound claudin-1 and claudin-5 is likely the cause for the observed drop in electrical resistance and heightened paracellular leakage (Fig. 3). Interestingly, in similar fashion as METH, mephedrone acts on endothelial expression of claudin-5 both in vivo [21] and in vitro [19, 21, 38].

In the current study, we also examined the level and subcellular distribution of occludin which is another key protein of the tight junctional complex and is expressed at higher levels in brain than non-cerebral vasculature. Occludin is not a critical structural component of the tight junctions as overexpression of occludin or its depletion does not affect the number or the morphology of tight junctions [44–47]. Unlike claudins, occludin does not impact the biogenesis of tight junctions but rather is involved in the fine tuning of permeability [44, 45]. Occludin is also critically involved in the regulation of BBB function in response to cytokines [48]. Post-translationally modifications of occluding were shown to modulate the fencing and paracellular permeability of the tight junction complex [49]. Here, we found that neither cytosolic nor membrane-bound fraction of occludin was significantly affected by mephedrone (Fig. 3). This observation suggests that mephedrone negatively affects paracellular barrier function via structural changes (i.e., claudin-5) in contrast to a more modulatory means (occludin). It is important to note that intracellular pathways that affect claudin expression/stability do not necessarily affect occludin in a similar way or vice versa. Indeed, although both types of proteins are part of the tight junction complex, their regulation differs. Certainly, these findings warrant further study since it suggests that cathinones initiate divergent intracellular signaling events that affect members of the tight junction complex differently.

We next evaluated the possibility of proinflammatory activation of endothelial cells upon mephedrone exposure. Several reports suggested that under the effect of insult, such as ischemia or substance abuse, the brain endothelium begin to release proinflammatory cytokines, particularly IL-1β [18, 23, 24]. To compare with previous findings on METH, which was reported to upregulate expression of proinflammatory cytokine both in vitro and in vivo [18, 21], we analyzed cytokine concentrations in the cell culture medium of mephedrone-treated endothelial cells. Here, we report that mephedrone heightens the secretion of proinflammatory cytokines-1β, IL-2, IL-8, IL-13, and TNFα. Interestingly, IL-2 was reported to regulate port-translational modifications of epithelial tight junction proteins [50], and in this report, we indeed observe higher molecular weight occludin that most like represents hyperphosphorylated form (Fig. 3a).

Cerebrovascular endothelial cells activation and resulting vascular leakage are considered to be the key initial steps in the process of neuroinflammation. PECAM-1 is a well-characterized multifaceted cell adhesion molecule which has been implicated in the regulation of vascular permeability by modulating the structure and function of tight junctions and transendothelial migration of immune cells in response inflammatory challenge. Vascular inflammation mediated by PECAM-1 is triggered by IL-1β and is not only dependent on NF-κB activity but also regulates NF-κB expression itself [51]. Here, we report that extracellular PECAM-1 was transiently increased in hBMVEC at 2 h after mephedrone application. It is likely that the upregulated extracellular localization of PECAM-1 facilitates transendothelial migration efficient immune cells in vivo. This finding aligns with our results of upregulated IL-1β and IL-8 (which is potent chemotactic factor) secretion (Fig. 4d) and activation NF-κB signaling in mephedrone-treated hBMVECs (Fig. 6).

Matrix metalloproteinases are enzymes involved in the remodeling of extracellular matrix under physiological and pathological conditions. Among other family members, MMP3, MMP9, and MMP12 are of particular interests due to their vast involvement in BBB pathology [32, 52]. Thus, MMP9 is known to induce enhanced phosphorylation and subsequent degradation of TJ proteins: occludin, ZO-1, and claudin-5 [52, 53] particularly in response to METH and MDMA [21, 22]. VEGF-A and proinflammatory cytokine IL-13 (both were elevated in our study) are factors that positively induce MMPs transcriptional regulation. Here, we report that mephedrone treatment not only increases *MMP3* and *MMP9* mRNA expression but also upregulates MMP9 enzymatic activity providing further evidence for the impact that cathinones may have on the dysregulation of the BBB.

Next, we explored the possibility that mephedrone may be inducing angiogenic program of brain endothelial cells. Elevated expression of angiogenic factors VEGF-A and PDFGF-BB was implicated to mediate vascular leakiness in substance abuse [35, 36, 54, 55]. Surprisingly, we found that mephedrone induces excessive secretion of VEGF-A and PDGF-BB in hBMVEC (Fig. 5), while cell proliferation kinetics was not affected (Fig. 1b). Overall, upregulated secretion of angiogenic growth factors, proinflammatory cytokines, and MMPs that we observed in this study resembles the description of inflammaging [56], and future investigation are needed to shed light whether mephedrone accelerates the process of biological aging of the cerebral endothelium and worsen age-related dysfunctions of the BBB.

NF-κB is a transcription factor activated by large number of signals like pathogens, stress, ethanol, and drugs of abuse [57, 58] and is considered to be a central component of the innate immune response and neuroinflammation. MMPs, inflammatory cytokines, and cell adhesion molecules are all under the transcriptional control of NF-κB [57]. Although, there are five subunits (p65, p52, p50, RelB, and c-Rel) that can combine to assemble NF-κB active dimer, only p65, RelB, and c-Rel have transactivation potential. The two most common dimers are p65/p50 heterodimer being the activator of transcription and p50/p50 homodimer being the repressor of transcription [58]. Activation of NF-κB and its contribution to BBB disruption occurs under a range of inflammatory insults and substance abuse [32, 57, 58]. Thus, cathinone and khat extract were reported to suppress (former) or induce (latter) phosphorylation of NF-κB in in vitro culture of leukocytes [59] supporting our findings in brain endothelial cells. To further understand how mephedrone may be activating NF-κB, we explored TLR4 signaling which has been reported to mediate METH-induced activation of NF-κB [37]. Inhibition of TLR4 abolished NF-κB activation, thus revealing the upstream signaling molecules that mediate the effects of mephedrone.

## Conclusion

We are the first to report that mephedrone-induced damage of the BBB involves alterations in tight junction protein complex, barrier leakiness or hyperpermeability, vascular endothelial activation, and inflammation. Therefore, it is possible that the vasogenic cerebral edema and neuroinflammation often seen in clinical cases of cathinone overdose result from the imbalanced functionality of the brain endothelial cells. Additionally, in the case of comorbidities such as HIV, mephedrone could exacerbate NeuroAIDS by facilitating migration of HIV-infected immune cells through the BBB into the brain parenchyma.

## Data Availability

All data generated or analyzed during this study are included in this manuscript.

## Abbreviations

ANOVA: Analysis of variance
BBB: Blood-brain barrier
DAPI: 4′,6-Diamidino-2-phenylindole
DEAE: Diethylaminoethyl
ECIS: Electric cell-substrate impedance sensing
ECM: Extracellular matrix
FBS: Fetal bovine serum
FITC: Fluorescein isothiocyanate
HIV-1: Human immunodeficiency virus 1
hBMVEC: Human brain microvascular endothelial cell
ICAM-1: Intercellular adhesion molecule 1
IL: Interleukin
MDMA: 3,4-Methylenedioxymethamphetamine
MDPV: Methylenedioxypyrovalerone
MMP: Matrix metalloproteinase
NaK-ATPase: Sodium-potassium adenosine triphosphatase
NPS: Novel psychoactive substance
NF-κB: Nuclear factor kappa B
NeuroAIDS: Neurological complications of acquired immunodeficiency syndrome
RNA: Ribonucleic acid
PECAM-1: Platelet endothelial cell adhesion molecule
PBS: Phosphate buffer saline
qPCR: Quantitative polymerase chain reaction
PDGF-BB: Platelet-derived growth factor subunit BB
PP: Paracellular permeability
SEM: Standard error of mean
TEER: Transendothelial electrical resistance
TIMP-1: Tissue inhibitor matrix metalloproteinase 1
TGF-β: Tumor growth factor beta
TNFα: Tumor necrosis factor alpha
TLR-4: Toll-like receptor 4
VEGF-A: Vascular endothelial growth factor A
VCAM-1: Vascular cell adhesion molecule 1
ZO-1: Zona occludens 1
